# Fluorescence In Situ Hybridization Analysis of Atypical Melanocytic Proliferations and Melanoma in Young Patients

**DOI:** 10.1111/pde.12382

**Published:** 2014-06-13

**Authors:** Emilia H DeMarchis, Susan M Swetter, Charay D Jennings, Jinah Kim

**Affiliations:** * School of Medicine, Stanford UniversityStanford, California, USA; † Pigmented Lesion and Melanoma Program, Department of Dermatology, Stanford University Medical CenterStanford, California, USA; ‡ Veterans Affairs Palo Alto Health Care SystemPalo Alto, California, USA; ¶ Department of Pathology, Stanford University Medical CenterStanford, California, USA

## Abstract

Morphologic heterogeneity among melanocytic proliferations is a common challenge in the diagnosis of melanoma. In particular, atypical melanocytic lesions in children, adolescents, and young adults may be difficult to classify because of significant morphologic overlap with melanoma. Recently a four-probe fluorescence in situ hybridization (FISH) protocol to detect chromosomal abnormalities in chromosomes 6 and 11 has shown promise for improving the classification of melanocytic lesions. We sought to determine the correlation between FISH results, morphology, and clinical outcomes in a series of challenging melanocytic proliferations in young patients. We retrospectively performed the standard four-probe FISH analysis on 21 melanocytic neoplasms from 21 patients younger than 25 years of age (range 5–25 years, mean 14.6 years) from Stanford University Medical Center who were prospectively followed for a median of 51 months (range 1–136 months). The study cohort included patients with 5 confirmed melanomas, 2 melanocytic tumors of uncertain malignant potential (MelTUMPs), 10 morphologically challenging atypical Spitz tumors (ASTs), and 4 typical Spitz nevi. FISH detected chromosomal aberrations in all five melanomas and in one MelTUMP, in which the patient developed subsequent lymph node and distant metastasis. All 10 ASTs, 4 Spitz nevi, and 1 of 2 MelTUMPs were negative for significant gains or losses in chromosomes 6 and 11q. Our findings demonstrated a strong correlation between positive FISH results and the histomorphologic impression of melanoma. This finding was also true for the MelTUMP with poor clinical outcome. Therefore FISH may serve as a helpful adjunct in the classification of controversial melanocytic tumors in young patients.

Melanoma is rare in children, accounting for only 7% of cancers in patients ages 15 to 19 years 1. As with adult melanoma, tumor thickness, ulceration, lymph node involvement, and advanced stage are negative survival indicators 2. Small sample size and inadequate clinical follow-up have resulted in limited guidelines for the diagnosis and classification of melanocytic tumors in young patients 3,4. Atypical melanocytic lesions are also challenging to diagnose in younger age groups, with a higher incidence of atypical Spitz lesions 5–7. This difficulty in analyzing atypical melanocytic proliferations was highlighted recently in an evaluation of an international registry for pediatric melanoma and atypical melanocytic proliferations 1.

Delayed diagnosis of pediatric melanomas may result in greater mortality 1 and poorer long-term survival in children with localized (90%) versus widespread (60.1%) disease 6. Misdiagnosis of melanocytic lesions occurs with 40% of conventional Spitz tumors in children overdiagnosed as melanomas 8. In addition, 14% of melanocytic nevi and 11% of melanomas were misdiagnosed 9. High interobserver variability is a well-documented occurrence in these atypical melanocytic lesions. Therefore early and accurate diagnosis of melanoma is critical to improve survival and prevent unnecessary surgery, demonstrating the importance of using molecular techniques for diagnosis.

Fluorescence in situ hybridization (FISH) protocols to detect abnormalities in chromosomes 6 and 11 are reported to improve the classification of melanocytic lesions and to provide prognostic information in atypical lesions 10,11. A four-probe FISH panel (6p25, centromere 6, 6q23, and 11q13), originally established by Gerami et al 12, has been used to analyze a range of melanocytic lesions, with more than 80% of known melanomas demonstrating positivity in the FISH panel 12, 34.7% of atypical Spitz tumors (ASTs) demonstrating negativity 13, and 15% of benign Spitz nevi demonstrating positivity for a gain in 11p 14, but current studies have not focused on a young patient population.

There is increasing interest in the histopathologic diagnostic criteria and long-term outcomes of melanocytic lesions in younger age groups 3–5, and FISH provides a potential means of improved classification. Children have been included in FISH studies 10,14, but direct analysis of FISH results in this age group for typical and atypical melanocytic lesions has not been performed. We sought to determine the correlation between four-probe FISH results, morphology, and clinical outcomes in a series of challenging melanocytic proliferations in young patients with long-term prospective follow-up.

## Materials and Methods

**Figure 1:**
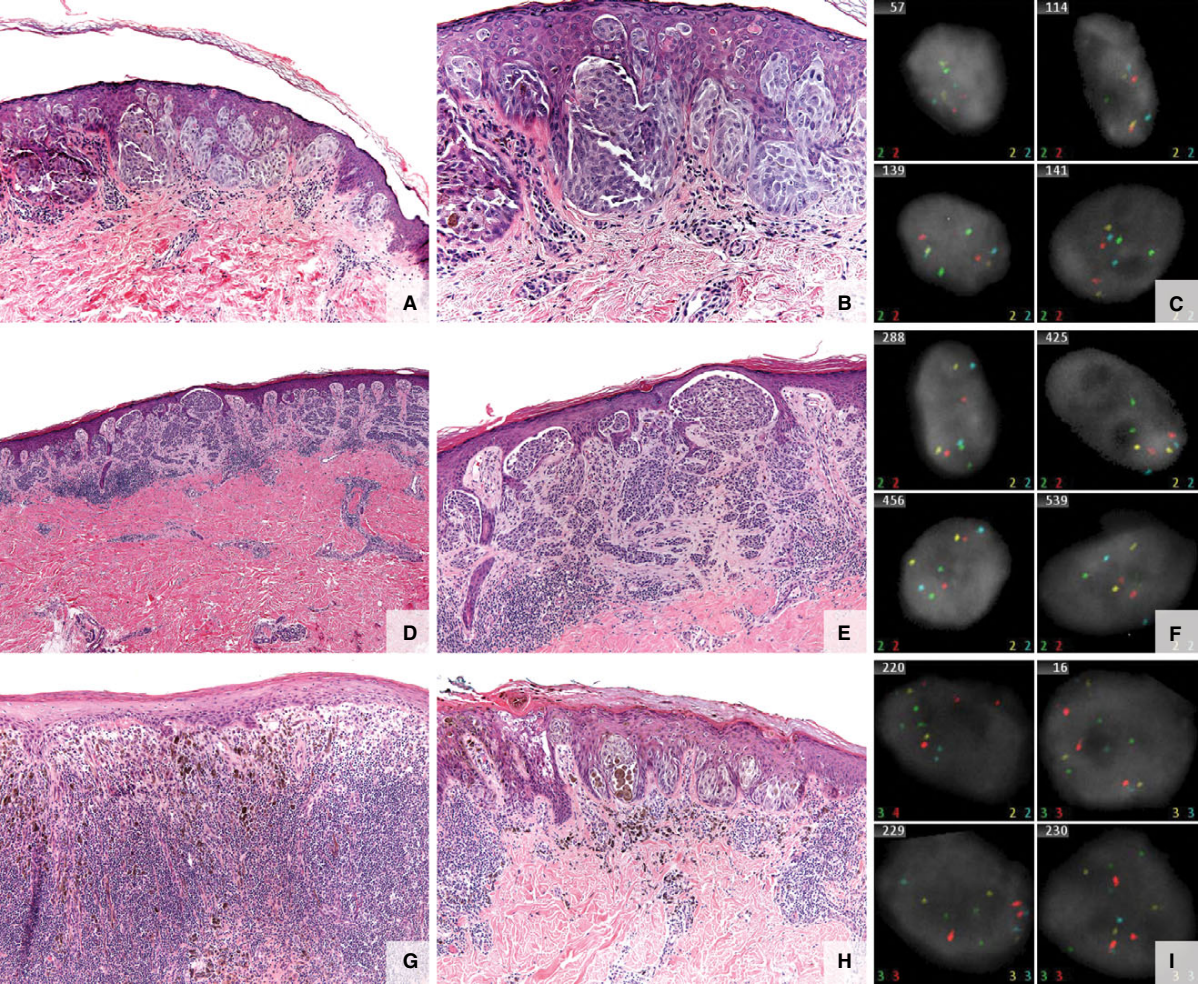
Spitz/Spitzoid lesions. (A–C) Specimen from a 10-year-old boy with a 4-mm brown papule on the left upper arm. (A, B) Spitz nevus—small, well-circumscribed, symmetric junctional melanocytic lesion composed of vertically oriented nests of spindled to epithelioid melanocytes with abundant cytoplasm, large nuclei, prominent nucleoli, and prominent pagetoid scatter of the melanocytes. (C) Fluorescence in situ hybridization (FISH) negative. (D–F) Specimen from a 25-year-old woman with a right lower leg lesion. (D, E) Atypical Spitz tumor. Compound melanocytic proliferation, with spindle features compatible with a Spitz nevus. Cytologic features vary from larger discohesive cells with prominent nucleoli to smaller mature melanocytes in the dermal component. Mild inflammatory infiltrate associated with this lesion and occasional epidermal Kamino bodies. No significant pagetoid spread or dermal mitotic figures. Hyperchromasia of dermal melanocytes, dyshesion within epidermal nests, and lack of definitive dermal architectural and cytologic maturation are seen. (F) FISH negative. (G–I) Specimen from a 16-year-old boy with a dark brown macule with central black papule, slowly enlarging. (G, H) Spitzoid melanoma. Atypical but well-defined compound melanocytic lesion, asymmetric with heavy, brisk lymphocytic host response and uneven pigmentation. Prominent, multifocal pagetoid upward scatter of malignant melanocytes into the epidermis. The tumor expands the dermis and focally consumes the epidermis. The atypical melanocytes are epithelioid with pink–brown cytoplasm and pleomorphic, irregular nuclear contours. Frequent mitotic figures are identified and enumerated at 3/mm^2^. (I) FISH positive (*RREB* and *CCND1* positive) of uncertain malignant potential.

Following institutional review board approval, the four-probe FISH panel was retrospectively performed on a series of atypical melanocytic neoplasms in patients age 25 years and younger who were identified and have been prospectively followed in the Stanford Pigmented Lesion and Melanoma Clinic/Lucile Packard Children's Hospital pediatric dermatology clinics from April 1999 through December 2013, as previously described 15. Cases diagnosed from April 1999 to November 2012 with available formalin-fixed, paraffin-embedded (FFPE) blocks were reviewed for four different categories: Spitz nevi, ASTs, melanocytic tumors of uncertain malignant potential (MelTUMPs), and melanomas. Although the majority of our MelTUMPs were of the spitzoid type, two lesions had features of pigmented epithelioid melanocytoma, and thus we elected to use this term rather than Spitzoid tumors of uncertain malignant potential (Fig.1) or ASTs, which have also been used to describe these challenging lesions. In contrast to the melanomas, complete or focal maturation with increasing dermal depth were more commonly seen in the MelTUMPs 15. After review by two dermatopathologists (JK, CDJ), 29 cases were included, but 5 lacked adequate tissue for FISH analysis.

**Figure 2:**
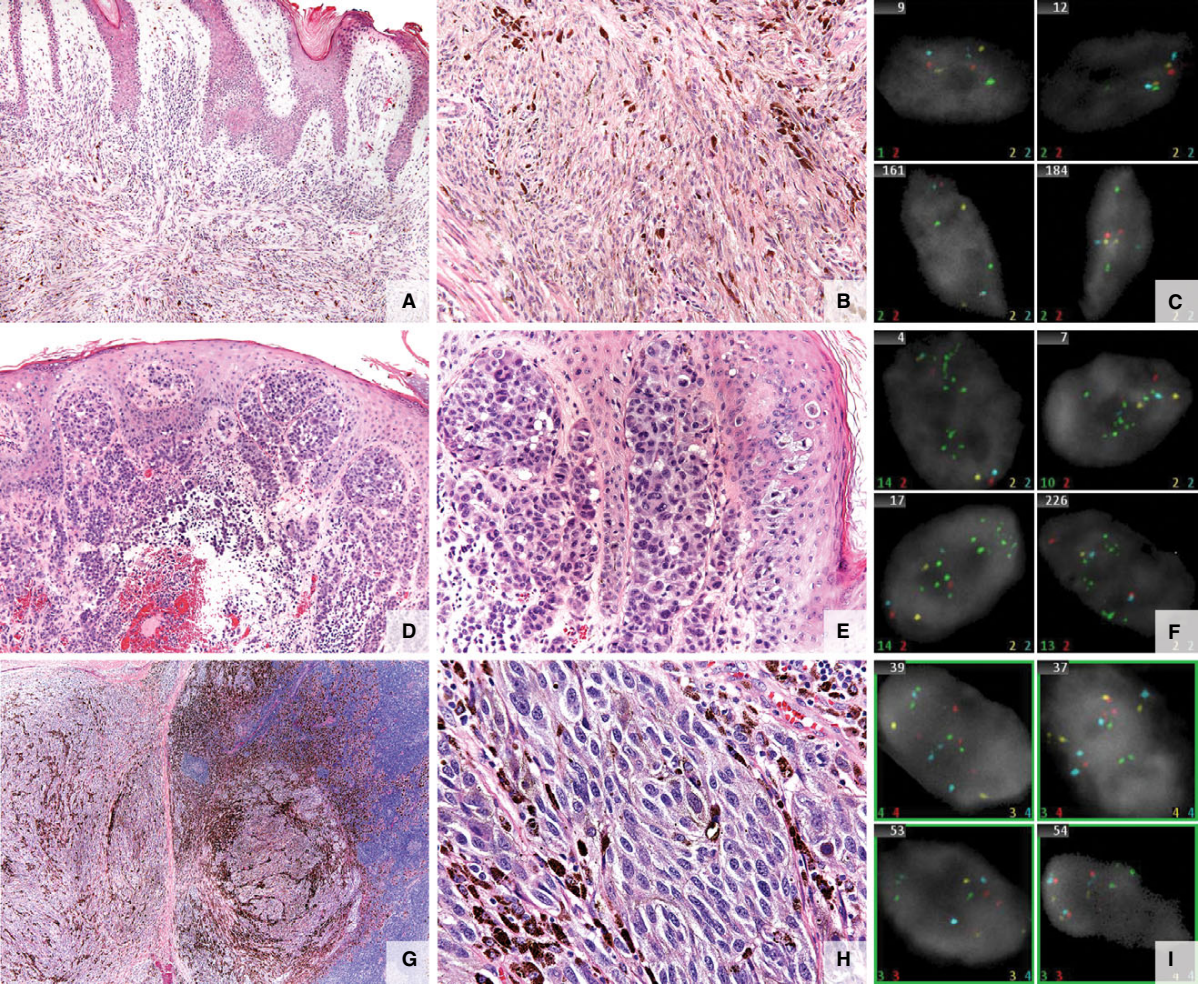
Atypical melanocytic lesions. (A–C) Specimen from a 20 year-old man with a left posterior scalp lesion. (A, B) Atypical melanocytic tumor of uncertain malignant potential (MelTUMP). Infiltrative predominantly intradermal spindle cell melanocytic proliferation extending to subcutaneous tissue. The epidermis is irregularly acanthotic without evidence of ulceration. Composed of spindled melanocytes in intersecting fascicles. A dermal mitotic figure is identified on hematoxylin and eosin stained sections. Melan-A and Ki-67 double-labeled immunohistochemical stain failed to identify a proliferating dermal melanocytic population. (C) Fluorescence in situ hybridization (FISH) negative. (D–F) Specimen from a 16-year-old girl with scalp melanoma. (D, E) Melanoma. Extensive epidermal ulceration and a markedly atypical compound melanocytic population of lentiginous, haphazardly nested spindled melanocytes with dusky cytoplasm, pleomorphic nuclei, and prominent nucleoli within the epidermis with extensive pagetoid scatter. Kamino bodies are not appreciated. Atypical cells extending from the epidermis through the dermis into the subcutaneous fat demonstrate numerous mitotic figures (5/mm^2^) and lack maturation with downward descent. There is ulceration and prominent epidermal consumption by the melanocytic proliferation. Focally, areas suggestive of regression are noted at the epidermal surface of the lesion. (F) FISH positive (*CCND1* positive). (G–I) Specimen from a 15-year-old boy with an enlarged mastoid lymph node. The patient had an initial atypical compound melanocytic proliferation of the right scalp diagnosed at 13 years of age. (G, H) Metastatic melanoma. Lymph node extensively infiltrated by an array of atypical pigmented cells with high nuclear to cytoplasmic ratios, irregular nucleoli, and dense chromatin. Heavy pigmentation is present in some of the cells. Lesional cells are mitotically active and zones of necrosis and areas of fibrosis are seen. (I) FISH positive (*RREB* and *CCND1* positive).

Relevant sections on the hematoxylin and eosin (H&E) slides for the 24 cases for FISH were reviewed and pertinent regions outlined. The study cases were then blinded and submitted for testing (Neogenomics Laboratories, Irvine, CA) using the standard four probes for melanoma. Probes consisted of *RREB1* (6p25), *CEN6* (centromere 6, *MYB* (6q23), and *CCND1* (11q13). According to the FISH protocol at the laboratory, the probe signal cutoff values used were 53% *RREB1:CEN6*, 42% *MYB:CEN6*, 16% *RREB1*, and 19% *CCND1* > 2, resulting in 95% specificity and 84% sensitivity (Fig.2) (Table1) 11,16.

**Table 1:** Probe Construction

Gene	Loci	Probe color	Abnormal	Abnormality	Cutoff%*
*RREB1*	6p25	Red	>2 red	Aneuploidy of *RREB1*	16
*RREB1* *CEN6*	6p25 CEN6	Red Blue	red > aqua	Aneuploidy of *RREB1*	53
*MYB* *CEN6*	6q23 CEN6	Yellow Blue	yellow < aqua	Deletion of *MYB*	42
*CCND1*	11q13	Green	>2 green	Aneuploidy of *CCND1*	19

* Interpret in the context of other pathology and clinical data.

Clinical features and outcome data were collected and histopathologic analysis of the study cases was performed. Data included age, sex, site of lesion, Breslow depth and staging for melanomas, therapy rendered, date of follow-up, and long-term clinical outcome. Correlations between histopathologic analysis, FISH results, and clinical outcome were evaluated. All statistics were performed using Stata version 11 (StataCorp, College Station, TX).

## Results

### Patient Demographics

Of 24 cases submitted for FISH, 3 were excluded because FISH results were inconclusive (87.5% efficacy). The remaining 21 melanocytic lesions were from 21 patients with a median age at diagnosis of 16 years (range 5–25 years, standard deviation [SD] 6.26). Fifteen cases had long-term follow-up (≥1 year), with a median 60 months of follow-up overall (range 1–136 months, SD 37.3). There were 11 boys and 10 girls, with similar male-to-female ratios for benign and malignant disease (7:7 and 4:3, respectfully). Girls were, on average, younger than boys (13.7 vs 15.4).

### Tumor Characteristics

There were 4 typical Spitz nevi, 10 morphologically challenging ASTs, 2 MelTUMPs, and 5 confirmed melanomas. Of the Spitz nevi, three were compound and one was junctional. Six of the ASTs were compound and one was intradermal. All patients with ASTs and Spitz nevi with follow-up of 1 year or more (9/14, 63.3%) were disease-free after an average of 64.2 months; two patients with typical Spitz nevi and two with ASTs were not followed over the long term in the pediatric dermatology clinics after biopsy or excision (range 1–9 months). Of the ASTs, one was from a patient with an agminated Spitz nevus within a nevus spilus. Two other ASTs were diagnosed as invasive melanoma and MelTUMP at an outside hospital. For the lesion originally treated as an invasive melanoma, the patient had undergone a sentinel lymph node biopsy (SLNB) (positive for metastasis) and a completion lymph node dissection (CLND) (negative for metastasis), but had declined interferon treatment and was clinically followed using serial positron emission tomography and computed tomography. The patient was originally diagnosed with a MelTUMP with residual disease treated using wide local excision (WLE) and SLNB, which was negative.

Of the two MelTUMPs diagnosed and treated at Stanford University Medical Center, one was intradermal and one was compound. The patient with the compound MelTUMP, which had features of pigmented epithelioid melanocytoma in the primary tumor, developed regional lymph node metastasis 2 years after WLE and negative SLNB, with subsequent additional regional nodal and distant pulmonary metastasis another 2 years later. The other patient with MelTUMP was lost to follow-up.

The mean Breslow thickness of the five melanomas was 2.3 mm (range 0.85–5.25 mm). Histologic subtypes included one spitzoid, one nevoid, and three superficial spreading (two with ulceration). WLE and SLNB were performed in all patients with melanoma, with SLNB positive in one patient who died from metastatic disease 15 months after diagnosis. Sentinel lymph node biopsy was negative in the other four patients with melanoma, although one developed subsequent regional nodal and in-transit recurrence followed by distant nodal and pulmonary metastasis. Three of the patients with melanoma had no evidence of disease after a mean of 83.3 months of follow-up (range 60–98 months, SD 20.4) (Table2).

**Table 2:** Patient Demographic Characteristics and Follow-Up

Histopathologic diagnosis	Age (years)	Sex	Site	Therapy	Clinical follow-up	Follow-up (months)
Junctional SN	10	Male	Left upper arm	Excised	NED	106
Compound ST	8	Female	Right arm	Excised	NED	51
Compound ST	8	Female	Right inner knee	Excised	NED	9
Compound ST	7	Female	Right lower leg	Transected, NFT	NED	1
Predominantly intradermal AST from agminated SN	22	Male	Midback	Excised, negative SLNB	NED	60
Severely AST	25	Female	Right lower leg	Excised	NED	136
Compound AST	23	Female	Right midback	Excised	NED	75
Borderline ST	9	Male	Posterior chest wall	Excised, negative SLNB	NED	12
Compound AST	8	Male	Right lateral knee	Excised	NED	97
AST	5	Female	Nose	Excised	NED	6
Compound AST	9	Female	Left thigh	Excised	NED	1
Compound AST	19	Male	Upper back	Excised	NED	9
Compound AST	23	Male	Left 2^nd^ toe	Excised, positive SLNB,† negative CLND	NED	28
Severely atypical compound spitzoid tumor	10	Male	Right knee	Transected, status post WLE, negative SLNB	NED	13
Predominantly intradermal AMP*	20	Male	Left posterior scalp	Transected (lost to follow-up)	NED	5
Compound AMP*	13	Male	Right mastoid LN (1^o^ scalp)	Excised, negative SLNB	LN‡ and distant metastases¶	56
Invasive SSM with ulceration	16	Female	Scalp	Excised, negative SLNB	LN and distant metastases	52
MM LN	20	Male	Right sup. inguinal LN (1^o^ right foot)	Excised, positive SLNB and CLND	LN and distant metastases, died of disease	15
SSM with ulceration	17	Female	Neck	Excised, negative SLNB	NED	98
Spitzoid melanoma	16	Male	Left upper back	Excised, negative SLNB	NED	92
SSM	19	Female	Left cheek	Excised, negative SLNB	NED	60

SN, Spitz nevus; ST, Spitz tumor; AST, atypical Spitz tumor; AMP, atypical melanocytic proliferation; SSM, superficial spreading melanoma; MM, metastatic melanoma; NED, no evidence of disease; NFT, no further therapy; CLND, complete lymph node dissection; WLE, wide local excision; NED, no evidence of disease; SLNB, sentinel lymph node biopsy; LN, lymph node.

* Treated as melanocytic tumor of uncertain malignant potential.

† SLNB positive for one node in left groin.

‡ LN metastases noted at 24 months.

¶ Distant metastases noted at 45 months.

### FISH Analysis

The four-probe FISH panel detected chromosomal aberrations in all five melanomas and in the one patient with MelTUMP who later developed regional lymph node and distant metastasis. Initial four-probe FISH on the primary tumor specimen of this patient (2 years before LN metastasis and 4 years before distant metastasis) was negative. All 10 ASTs, 4 Spitz nevi, and 1 of 2 MelTUMPs were negative for significant gains or losses in chromosomes 6 and 11q. The average age of the six patients with metastatic disease or positive FISH results was 17.3 years at the time of diagnosis, versus 13.2 years in patients with negative FISH and favorable outcome (Table3). Our patient with negative FISH (performed at an outside hospital) on biopsy developed FISH-positive macroscopic lymph node metastasis 2 years later.

**Table 3:** FISH Results for Four-Probe Panel, According to Diagnosis

Diagnosis	Age (years)	Sex	Clinical follow-up	Follow-up (months)	*RREB* > 2	*RREB1* > *CEN6*	*MYB* < *CEN6*	*CCND1* > 2
Junctional SN	10	Male	NED	106	Normal	Normal	Normal	Normal
Compound ST	8	Female	NED	51	Normal	Normal	Normal	Normal
Compound ST	8	Female	NED	9	Normal	Normal	Normal	Normal
Compound ST	7	Female	NED	1	Normal	Normal	Normal	Normal
Predominantly intradermal AST from agminated SN	22	Male	NED	60	Normal	Normal	Normal	Normal
Severely AST	25	Female	NED	136	Normal	Normal	Normal	Normal
Compound AST	23	Female	NED	75	Normal	Normal	Normal	Normal
Borderline ST	9	Male	NED	12	Normal	Normal	Normal	Normal
Compound AST	8	Male	NED	97	Normal	Normal	Normal	Normal
AST	5	Female	NED	6	Normal	Normal	Normal	Normal
Compound AST	9	Female	NED	1	Normal	Normal	Normal	Normal
Compound AST	19	Male	NED	9	Normal	Normal	Normal	Normal
Compound AST	23	Male	NED	28	Normal	Normal	Normal	Normal
Severely atypical compound ST	10	Male	NED	13	Normal	Normal	Normal	Normal
Predominantly intradermal AMP	20	Male	NED	5	Normal	Normal	Normal	Normal
Compound AMP	13	Male	LN* and distant metastases†	56	Positive	Normal	Normal	Positive
Invasive SSM with ulceration	16	Female	LN and distant metastases	52	Normal	Normal	Normal	Positive
MM LN	20	Male	LN and distant metastases, dead of disease	15	Positive	Normal	Normal	Positive
SSM with ulceration	17	Female	NED	98	Positive	Normal	Normal	Positive
Spitzoid melanoma	16	Male	NED	92	Positive	Normal	Normal	Positive
SSM	19	Female	NED	60	Positive	Normal	Normal	Positive

FISH, fluorescence in situ hybridization; SN, Spitz nevus; ST, Spitz tumor; AST, atypical Spitz tumor; AMP, atypical melanocytic proliferation; SSM, superficial spreading melanoma; MM, metastatic melanoma; NED, no evidence of disease; LN, lymph node.

* LN metastases noted at 24 months.

† Distant metastases noted at 45 months.

The FISH-positive MelTUMP and four of the melanomas (80%) showed aberrations in chromosomes 6 and 11, with aneuploidy of *RREB1* and *CCND1*. The remaining melanoma (with ulceration and eventual regional nodal distant metastases) showed aneuploidy in only *CCND1* (chromosome 11).

## Conclusions

Our study demonstrates the strong correlation between positive FISH results in melanocytic neoplasia in children and young adults ages 25 years and younger with morphologic features of melanoma, whereas negative FISH in patients with typical Spitz tumors and ASTs had a favorable long-term outcome. We focused our analysis on a group of controversial melanocytic neoplasms in which differentiation between malignant and benign was considered in 12 cases: 10 ASTs and 2 MelTUMPs. The negative FISH results from cases with a favorable outcome, including all typical Spitz nevi and ASTs, support the utility of this technique in the diagnosis and management of young patients with challenging melanocytic neoplasms. The clinical significance of FISH results may be helpful in ambiguous cases, such as MelTUMPs. Although this study was limited by a small sample size, FISH was able to identify chromosomal abnormalities in the patient with a MelTUMP who subsequently developed metastatic disease. Studies have shown that MelTUMPs and ASTs can metastasize, although there is much debate as to whether SLN metastasis, in particular, affects overall survival in these patients 16. Our cohort included one person with an AST that was treated at an outside hospital. The patient had a positive SLNB but negative CLND and no evidence of recurrent disease on subsequent follow-up, as well as negative FISH.

Five of six FISH-positive cases demonstrated aneuploidy of *RREB1* and *CCND1*, whereas one ulcerated melanoma with eventual LN and distant metastases demonstrated aneuploidy in only *CCND1*. Gerami et al previously showed that *CCND1* gains correlate with poor prognosis in cutaneous malignant melanoma 17, whereas *RREB1* has the greatest sensitivity for melanoma (*RREB1*, 72.9% vs *CCND1*, 20% for superficial spreading melanoma) 18. Likewise, *RREB1* and *CCND1* gains were found to be more frequently associated with aggressive ASTs 13, although the authors did not comment on the significance of having aneuploidy in one of the two, versus gains in both probes. In our series, the significance of the aggressive, ulcerated melanoma being selectively *CCND1* positive remains unclear, although it is an additional case supporting Gerami's finding of the aneuploidy correlating with poor prognosis.

Although studies have evaluated the utility of the four-probe FISH array in detecting the malignant potential of melanocytic lesions in older patients 10–12,19, there have recently been some studies using the 9p21 probe 19–21. Biallelic loss of 9p21 (p16) has been shown to correlate well with high-risk ASTs 13,20, with greater sensitivity and specificity for clinically aggressive ASTs when used in conjunction with the four-probe FISH array 19,21,22. In addition, ASTs with a homozygous 9p21 deletion more frequently demonstrated severe cytologic atypia, greater dermal mitotic activity, and clinical association of tumor extension beyond the sentinel lymph node than did ASTs with a heterozygous 9p21 deletion 23.

In our cohort, the average age was 13.7 years for the FISH-negative cases and 16.8 years for the FISH-positive cases. Using the two-sample *t*-test, the p-value was.16. Unfortunately, patient age was not significant in our cohort as an independent variable to predict positive FISH results. Larger multicenter studies may provide greater power to further evaluate whether age is useful in diagnostic algorithms.

Another molecular adjunct, comparative genomic hybridization (CGH), relies on detection of amplifications and deletions. Comparative genomic hybridization currently requires a larger tumor sample size with a high proportion of malignant cells for analysis and is unable to detect balanced translocations 24, which are some limitations for widespread clinical use. FISH can be performed on smaller samples with a smaller ratio of malignant to normal melanocytes 25, although that may be associated with a greater risk of false-positive results from tetraploidy 26. Conversely, FISH has the limitation of testing targeted specific chromosomal loci, whereas CGH can provide broader information. Some studies have shown better sensitivity for FISH than CGH in the analysis of controversial melanocytic neoplasms 14, whereas others have demonstrated the opposite 16. Additional genomic studies are required with long-term follow-up to better delineate the genes that are most helpful for diagnostic classification.

Single-nucleotide polymorphism (SNP) genomic microarrays (GMAs), which may require even less tissue that FISH, have recently been studied 26. The SNP analysis was 89% sensitive and 100% specific for melanoma, and although the study included Spitz nevi and MelTUMPs, there was no conclusive evidence that SNP-GMA is useful in identifying borderline lesions with malignant potential 26. Although lack of follow-up data and small sample size limited the study, it suggests the need for further analysis to determine the utility of SNP-GMA.

Our study limitations include small sample size, which is expected because of the rarity of atypical melanocytic lesions and melanomas in the pediatric and young adult population. A strength of our data is the long-term prospective follow-up, an average of nearly 4 years, allowing for clinical outcome correlation with FISH results. A larger, multicenter FISH analysis of atypical melanocytic lesions in the pediatric, adolescent, and young adult population would further validate our findings.

Although additional studies are needed to improve the diagnosis of controversial atypical melanocytic lesions in younger individuals, our study shows that FISH may be a helpful adjunct to current histopathologic diagnostic techniques. The utility of FISH as a complementary modality in the classification of melanocytic tumors in young patients warrants further study, although it appears clinically useful in younger populations with challenging melanocytic neoplasms.
